# Dominating role of crystal structure over defect chemistry in black and white zirconia on visible light photocatalytic activity

**DOI:** 10.1038/s41598-018-23648-0

**Published:** 2018-04-03

**Authors:** Sri Ramya Teeparthi, Eranezhuth Wasan Awin, Ravi Kumar

**Affiliations:** Laboratory for High Performance Ceramics, Department of Metallurgical and Materials Engineering, Indian Institute of Technology-Madras (IIT Madras), Chennai, 600036 India

## Abstract

Nanometric powder particles of white zirconia were synthesized through precursor route by the pyrolysis of zirconium (IV) butoxide at varied temperatures in air ranging from 900–1400 °C and were predominantly monoclinic in nature. To control the defect chemistry, the precursor was also pyrolyzed in a reduced atmosphere at 900 °C, eventually resulting in black zirconia. The stabilization of tetragonal phase and observed color change from white to black in samples pyrolyzed under reduced atmosphere was attributed to the creation of oxygen vacancies and disorder. The black and white zirconia produced delineated the influence of crystal structure and oxygen vacancies on the photocatalytic performance. Furthermore, zirconia synthesized at lower temperatures (600 and 800 °C) in air confirmed the detrimental role of tetragonal phase on the degradation behavior of methylene blue dye. High photocatalytic degradation rate for white zirconia was attributed to the presence of increased density of nano-sized pores and low recombination rate of electron-hole pairs as confirmed by PL measurements. Interestingly, black zirconia exemplified relatively limited activity albeit presence of oxygen vacancies. This negative effect was attributed to the presence of tetragonal phase and possibly, the insufficient creation of new energy states near valence and conduction band towards Fermi energy level.

## Introduction

Zirconia with a high negative conduction band potential is capable of generating holes with very strong oxidation power and currently being explored as a potential third generation photocatalyst. A high bandgap of 5.1 eV makes zirconia to be seldomly used in photocatalysis but the incorporation of metal ions has enhanced the efficiency of ZrO_2_ based catalytic systems. One of the earlier studies on the photocatalytic behavior of ZrO_2_ and Fe/ZrO_2_ semiconductors prepared by sol-gel technique was reported by S.G. Botta *et al*.^1^, in which the activity was compared in visible light with commercially available P-25. It was found that all the samples were active in this region but did not show appreciable catalytic activity in comparison with the commercially available P-25. Moreover, the incorporation of Fe (III) increased the activity of the catalyst, where, Fe (III) acted as electron or hole trap centers and the lower activity of ZrO_2_ in UV light was attributed to the intraband surface states. In recent times, nanocrystalline ZrO_2_ has been largely investigated for its potential in photocatalytic applications. Nano-porous zirconia electrospun fiber mats (NZEFM) by electrospinning using non-ionic F108 as a pore former was prepared by Niu *et al*.^2^. The high specific surface area and nano-porous “building block” structures which consisted of stacked zirconium oxide nanoparticles were reasoned out for the high photocatalytic activity reported for NZEFM. In an earlier study, the photocatalytic degradation of methylene blue by zirconia particles prepared by thermal plasma route have been correlated with the energy gap of monoclinic and tetragonal phases by Ashok B *et al*.^3^. Concomitantly, the photocatalytic activity of monoclinic ZrO_2_ has been attributed to the effect of oxygen-deficiency, porosity, high crystallinity and density of surface hydroxyl groups by Basahel *et al*.^4^. The photocatalytic degradation of organic dyes by tetragonal and monoclinic phases of zirconia either have been attributed to reduced particle size or oxygen vacancies or both. The band gap reduction due to doping with metal oxides and the synergistic effect of carbon has also been reported to aid the catalytic activity of zirconia in UV/visible light^5–15^.

In order to improve the catalytic activity and meet the need for new materials under visible light radiation, numerous studies have been carried out which includes introducing disorder in the surface layers. The visual appearance of the catalysts was found to be altering with the introduction of defects. It was reported that the disordered TiO_2_ crystals prepared by hydrogenation and one step reduction/crystallization process enhanced the solar photocatalytic activity in visible region^16–19^. Also in a recent study, Apurba *et al*.^20^, reported drastic improvement in the photocatalytic activity by inducing surface defects and oxygen vacancies in zirconia by the magnesiothermic reduction of monoclinic zirconia in reducing atmosphere.

The work done till now demonstrates the influence of particle size, defect concentration and crystal structure on the photocatalytic behavior of organic dyes both in UV and visible wavelength regions. A systematic study delineating the influence of each of these factors on the photocatalytic behavior of zirconia, however, is still lacking particularly in the visible wavelength region. Systematic and elegant experiments are required to understand some of the not so clearly understood scientific issues that can clearly limn the influence of crystal structure and defects on the photocatalytic degradation of organic dyes in visible light. Here, we report the synthesis and characterization of white and black zirconia by altering the pyrolysis atmosphere and temperature using a precursor route, thereby, controlling the crystal structure and defect chemistry. A comprehensive structural and spectro-chemical characterization accompanied by degradation studies elucidated with band gap measurements clarify clearly the role of crystal structures and defect chemistry on the photodegradation behavior of zirconia.

## Results and Discussion

### Thermal Properties

The TGA of zirconium butoxide clearly exemplifies three stages of mass loss (see supplementary data Figure S1). A huge mass loss of around 18.64% was seen from 25–250 °C which was attributed to the loss of water and hydroxyl groups^21^. The mass loss of 5.51% observed between 250–500 °C was accounted for evaporation of residual solvents, by-products and unreacted oligomers. The subsequent weight loss of 2.4% from 500–700 °C could be predominantly due to the decomposition reactions eventually resulting in the formation of ZrO_2_ particles. The endothermic peak around 950 °C was assigned to the phase transformation of t- ZrO_2_ to m-ZrO_2_, accompanied by a weight loss of 2.55% between 700–1300 °C^22^.

### Phase evolution of zirconia

**Figure 1:**
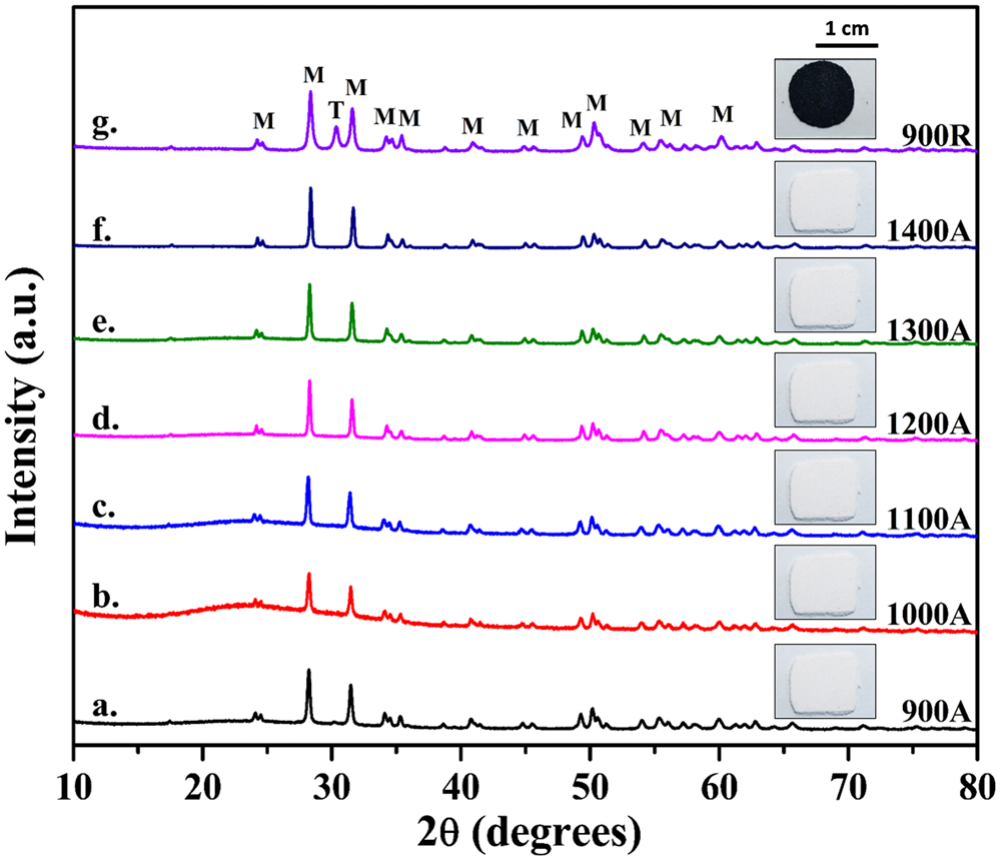
X-ray diffractograms of pyrolyzed zirconium butoxide; (**a–f**) in air from 900–1400 °C and (**g**) in reduced atmosphere at 900 °C.

X-ray diffractograms of zirconium butoxide pyrolyzed at varied temperatures are shown in Fig. 1(a–f) and the sample produced in reduced atmosphere is shown for comparison (Fig. 1g). In comparison with the ICSD data base (80050 and 97004), peaks observed at 24.2°, 28.2°, 31.4°, and 34.3° in Fig. 1(a–g) correspond to (100), (11 $\bar{1}$), (111) and (200) planes of monoclinic zirconia (space group: p 1 21/c 1) and the peak observed at 30.3° in Fig. 1g corresponds to the (011) plane of tetragonal zirconia (space group: p 42/n m c). All the samples processed in atmospheric ambience exhibited monoclinic phase, except for an extremely low intensity tetragonal peak (2θ = 30.3°) for **900A** sample. A distinct peak corresponding to tetragonal phase was observed for the **900R** sample indicating the influence of processing atmosphere on the retention of the tetragonal crystal structure. Rietveld analyses of the XRD data using FullProf Suite yielded tetragonal phase fractions of approximately 1% for **900A** and around 11% for **900R** samples (with chi- square values of 4.22 and 2.90 for **900A** and **900R** respectively). The Rietveld analyses data is provided in supplementary information (Figure S2).

**Figure 2:**
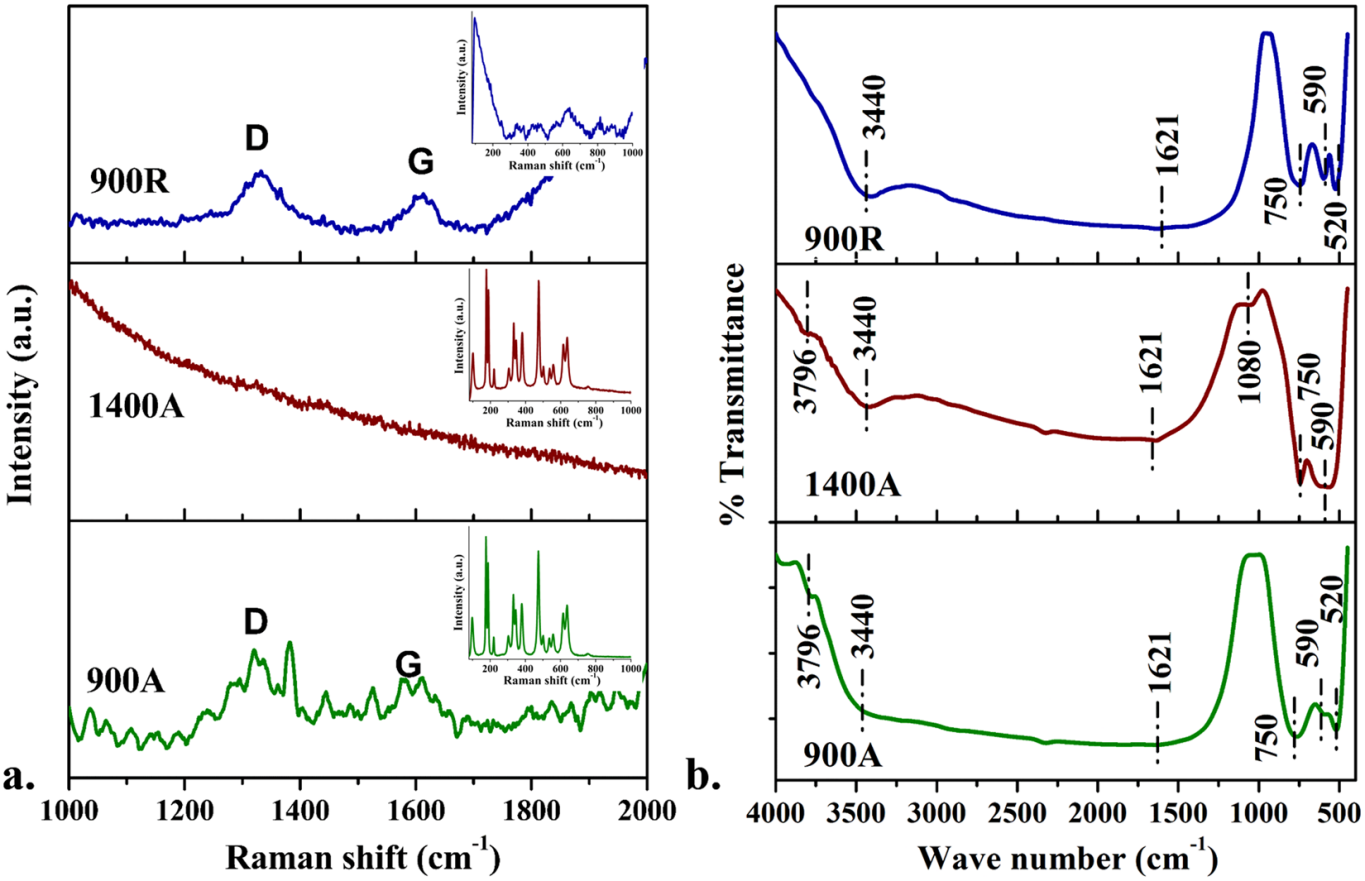
(**a**) Raman spectra of samples showing the presence of free carbon in the matrix; the insets in the graphs represents the peaks of monoclinic peaks and disordered surface; (**b**) Fourier transform infrared spectra of samples indicating bands of various functional groups.

Figure 2a shows the Raman spectra of **900A**, **1400A** and **900R** samples indicating the presence of free carbon for **900A** and **900R** samples exemplified through the D and G bands. It is clear that **1400A** sample obviously is free of carbon indicating that pyrolysis at 1400 °C in air removed free carbon completely from the material. The insets Fig. 2a show Raman spectra of the respective samples showing the confirmation of the presence of monoclinic phase. On the basis of factor group analysis, it has been shown that monoclinic phase with 18 (9Ag + 9Bg) and the tetragonal phase with 6 (A1g + B2g + 3Eg) were the Raman active vibrational modes^23^. **900A** and **1400A** has showed 14 sharp bands totally of monoclinic phase which were assigned as follows: A_g_ at 101, 177, 190, 306, 333, 346, 475, 558 and 637 cm^−1^; B_g_ at 221, 380, 501, 537 and 615 cm^−1^ ^4^. The peak intensity increased from 900 to 1400 °C and there was no much broadening or peak shift observed in the samples. The most intense peaks of tetragonal at 147 and 267 cm^−1^ were not observed in both the samples. However, the peaks centering at 476 and 637 cm^−1^ belongs to both monoclinic and tetragonal phase structures^24^. The **900R** (inset of Fig. 2a of **900R**) sample shows peak broadening and enormous decrease in the peak intensity. This may be due to the surface alteration or any oxygen vacancies caused by reduced atmosphere during reduction^20^. This supports the variation in color of the sample from white to black.

### Functional groups

The bands observed in the FT-IR spectra (Fig. 2b) of **900A**, **1400A** and **900R** near 3440 and 1621 cm^−1^ could be attributed to the stretching and bending vibrations of hydroxyl groups and adsorbed water molecules^25^. A weak band at around 3796 cm^−1^ refers to the confined OH groups^26^. A small peak near 1080 cm^−1^ was observed, possibly due to the vibrations of coordinated carbonate ions^27^. The sharp peak observed near 750 cm^−1^ corresponds to the vibrations of monoclinic zirconia and the peak around 590 cm^−1^ was attributed to the tetragonal zirconia. A strong band and a small weak band were observed in **900R** and **900A** at 590 cm^−1^. This confirms the presence of tetragonal phase in both the samples previously also detected in XRD. The bands observed from 750–520 cm^−1^ were assigned to the Zr-O vibrations produced from ZrO_2_^22^.

### Morphological features

**Figure 3:**
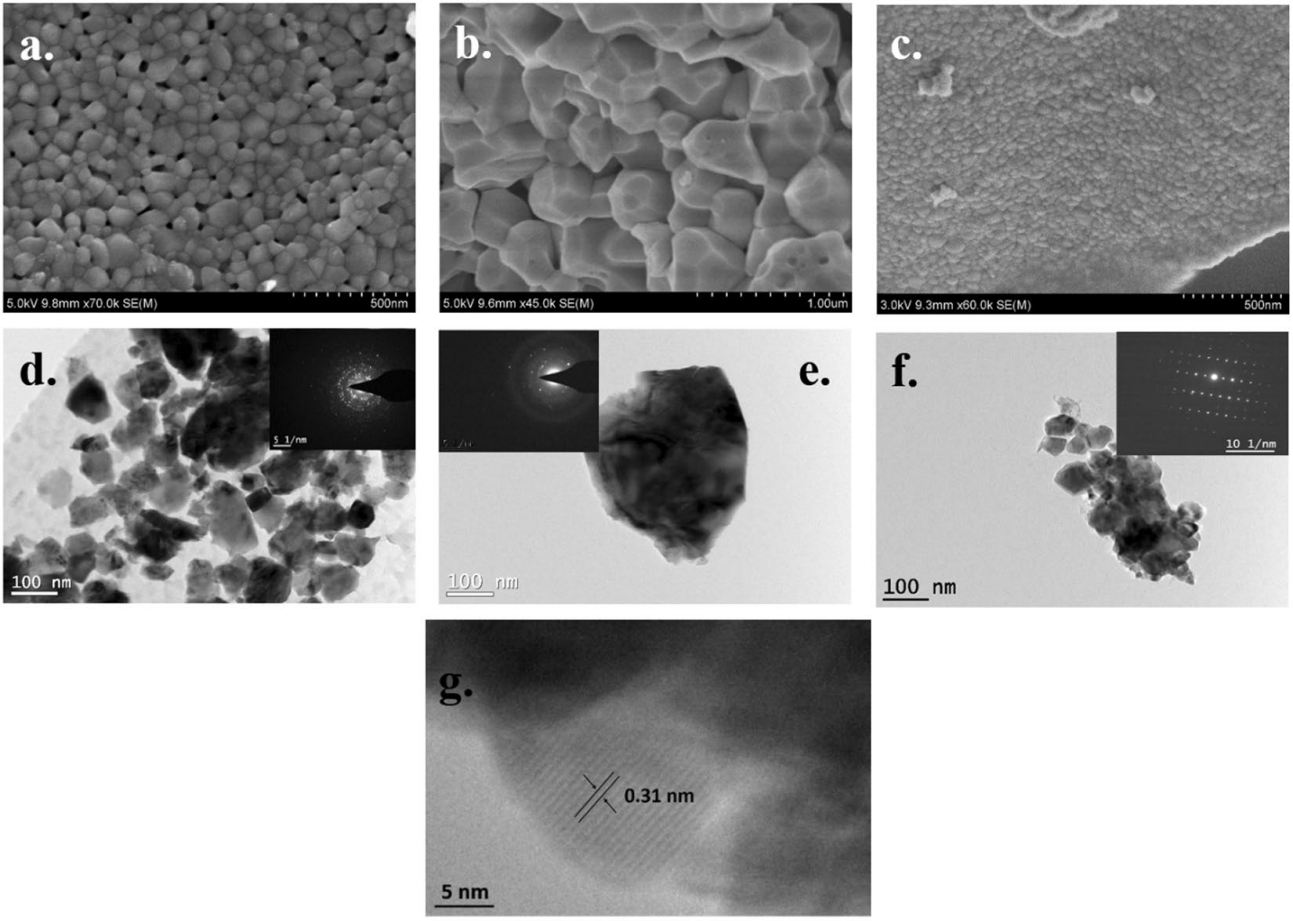
SEM micrographs (**a–c**) illustrating faceted features; TEM micrographs (**d–f**) with their respective SAED patterns **{**of (**a**,**d**) 900A, (**b**,**e**) 1400A, (**c**,**f**) 900R**}**; (**g**) HR-TEM image of 1400A showing fringes with spacing corresponding to the m-ZrO_2_.

Figure 3(a–c) exemplify the morphological features of as-pyrolyzed pulverized samples of **900A**, **1400A** and **900R** respectively using scanning electron microscopy. The average size of the crystals is in the order of ~70 nm for the **900A** sample (Fig. 3a). With increase in pyrolysis temperature, there is an obvious increase in the crystal size to around 0.4–0.5 μm as seen in Fig. 3b for the **1400A** sample. The faceted features of the monoclinic crystals can be clearly observed and one could expect the growth of these crystals with increase in temperature due to Ostwald ripening. However, in contrast to Fig. 3a and b, the sample pyrolyzed in reduced atmosphere showed crystal sizes less than 50 nm (Fig. 3c) indicating resistance to crystallization due to reduced oxygen partial pressure (p_o2_). Interestingly, in all the three cases, the pulverized particles seem to have partially sintered by solid state diffusion at the processing temperatures.

Further, the crystalline nature and crystallite size of the samples were confirmed by transmission electron microscopy. Figure 3(d–f) show the TEM micrographs of **900A**, **1400A** and **900R** samples. Using Image J software, the average crystallite size of the samples was calculated which ranges from 40–400 nm. The insets in the Fig. 3(d–f) represents the selective area electron diffraction (SAED) patterns of the pyrolyzed zirconia samples. The radius of the rings was measured and ratios between them correspond well to the ratios of the d-spacing of monoclinic structure of zirconia (ICSD-80048). In Fig. 3(d and f) the rings were the result of the polycrystalline diffraction from (111), (022), (002), (110), (001) and (113) planes of m-ZrO_2_. Figure 3e is a single crystal of zirconia which can be clearly understood from its SAED pattern exemplifying the spots. A set of well-defined parallel fringes with a spacing of 0.31 nm can be seen in Fig. 3g. This corresponds to the d-spacing of (11 $\bar{1}$) planes of monoclinic zirconia (ICSD-80048).

The nitrogen adsorption-desorption isotherm and textural properties of the pyrolyzed samples were depicted in Figure S3 and Table 1 (see supplementary information). The adsorption-desorption patterns belong to the type IV isotherm which indicates uniform size and mesoporous structure of the pyrolyzed samples. The BET surface area was determined to be 1.1095, 0.9512 and 0.639 m^2^/g for **900A**, **1400A** and **900R** samples respectively. The surface area was observed to be low and no significant change was observed between the samples. This shows a very little influence of surface area on the catalytic activity.

### Chemical states and surface defects

**Figure 4:**
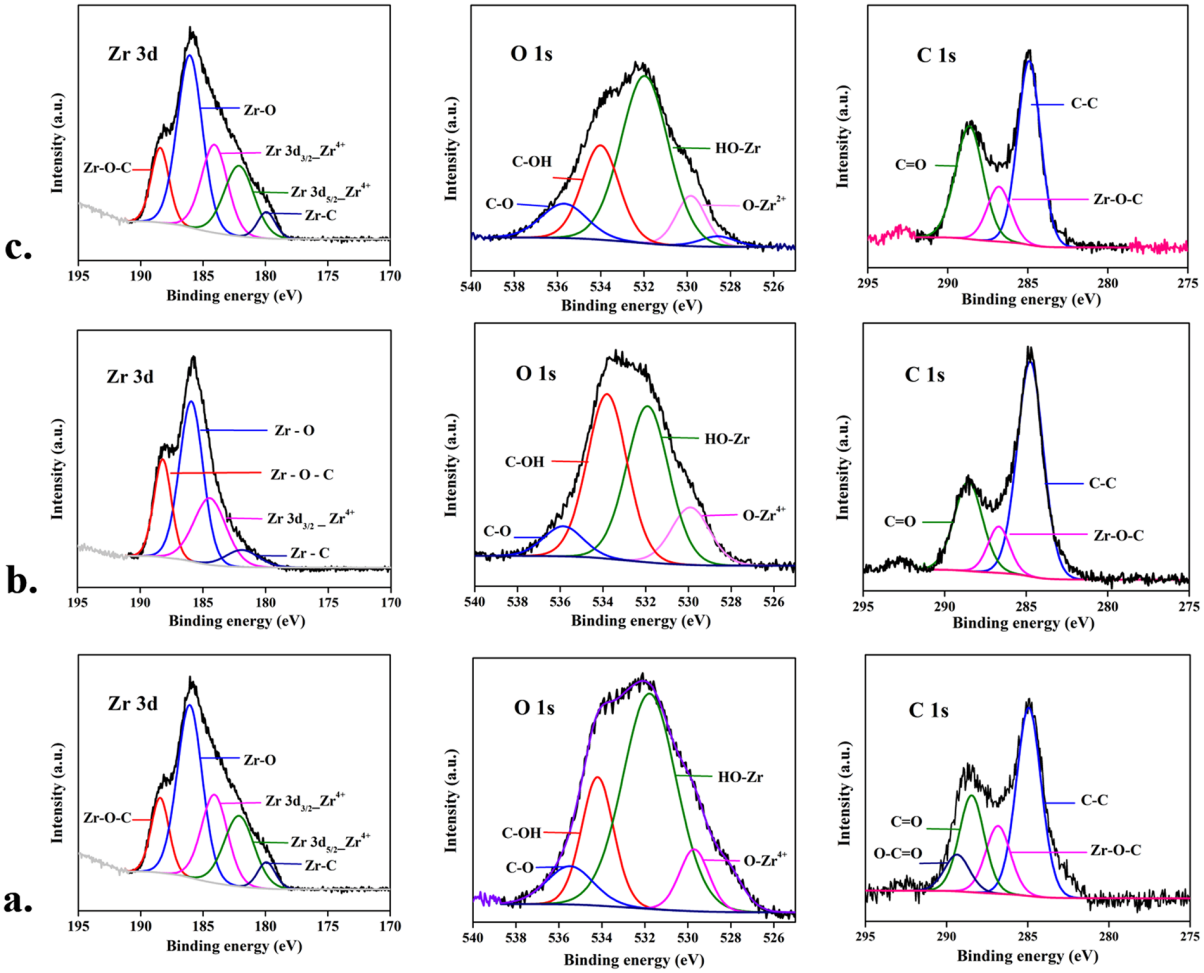
XPS spectra of Zr 3d, O 1 s, C 1 s spectra of (**a**) 900A, (**b**) 1400A and (**c**) 900R.

X-ray photoelectron spectroscopy confirmed the presence of surface defects, chemical state of Zr ions in ZrO_2_ and bonding between the elements present. Figure 4 shows XPS spectra containing different regions of **900A**, **1400A** and **900R** respectively. The binding energies at 532, 336 and 285 eV corresponds to the O 1 s, Zr 3p and C 1 s peaks of zirconia samples^28^ (see supplementary information Figure S4). In all these cases, the deconvolution of Zr 3d spectra resulted in two peaks at ~182 eV and ~184 eV which corresponds to the Zr 3d_5/2_ and Zr 3d_3/2_ respectively. Further analysis of Zr 3d spectra shows the evidence of reduced Zr species and Zr^(4-x)+^ ions. The values observed were in accordance with the values reported in literature^4^. The deconvolution of C 1 s peaks resulted in peaks corresponding to carbonyl carbon C=O (288.4 to 288.6 eV), aliphatic C-C (284.7 to 284.9 eV), Zr-O-C (286.6 to 286.8 eV) and of carboxylate carbon O-C=O (289.3 eV). There was an observance of shift in the peaks to higher binding energies in **900A** and **900R** which can be assigned to the charge transfer of Zr^4+^ to carbon matrix, thereby making the bond stronger^26^. The deconvolution of O 1 s spectrum resulted in the observation of Zr-OH, Zr-O, C-O and C-OH bonds with binding energies falling in the range of 531.7 to 531.9 eV, 529.7 to 529.9 eV, 534.2 to 535.8 eV and 533 to 534 eV, respectively. XPS results confirmed the presence of oxygen vacancies in the samples pyrolyzed in reduced atmosphere. The relative percentage of surface defects and the variation in intensity for Zr-OH and bonding of carbon to the matrix can be seen in Table 2 of supplementary data.

**Figure 5:**
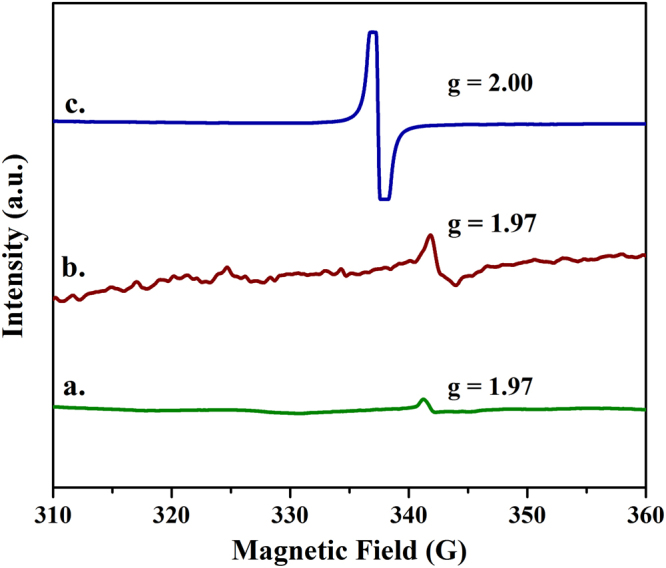
EPR analysis spectra of (**a**) 900A (**b**) 1400A and (**c**) 900R.

Electron paramagnetic resonance spectroscopy was used to confirm the presence of defects in the pyrolyzed samples. Figure 5 shows the EPR spectra of **900A**, **1400A** and **900R**. The EPR spectra of **900A** and **1400A** possess a signal corresponding to a **g** value of 1.97 which can be attributed to the free electron coming from Zr^3+^ species^29^. These signals were weak and can be understood that these were generated from the surface and not within the bulk of samples. The signal generated in the zirconia sample prepared in reduced atmosphere confirms the presence of oxygen vacancies with a **g** value of 2.00 attributing to the free radical coming from the oxygen vacancy^20^.

### DRS spectra and band gap measurements

**Figure 6:**
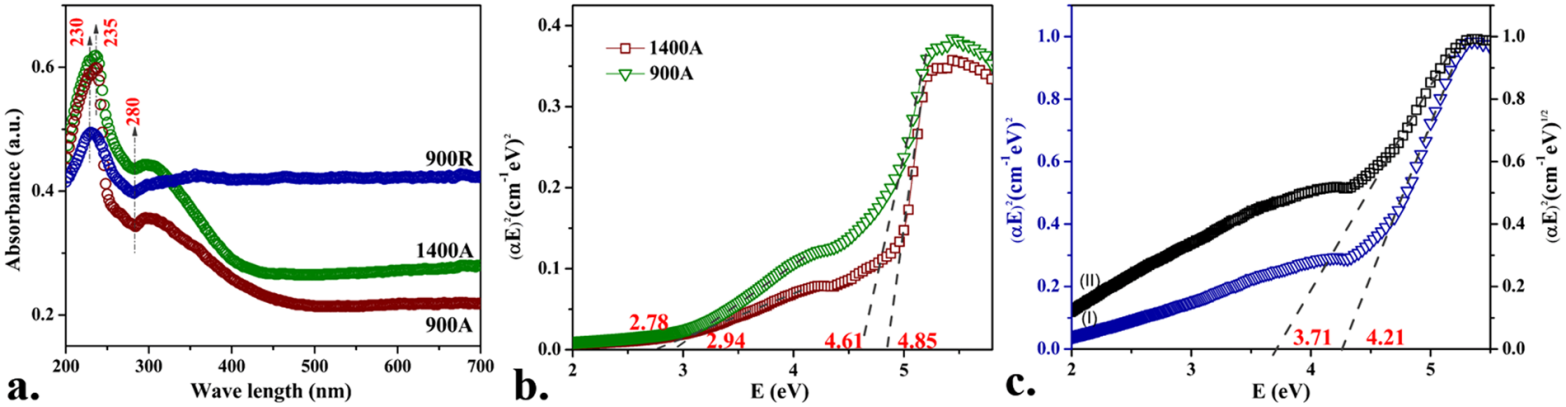
(**a**) UV-vis absorbance spectra of 900A, 1400A and 900R samples indicating various absorption peaks; Tauc plot obtained from absorption data extrapolating for optical bandgaps of (**b**) 900A and 1400A, (**c**) 900R.

Figure 6a shows diffuse reflectance spectra of **900A**, **1400A** and **900R** with an absorption range from 200–700 nm. A broad absorption in the range of 200–330 nm was observed in all the samples. The absorption peak around 235 nm was attributed to the photoexcitation from valence band to conduction band. In the case of **900A** and **1400A** samples with monoclinic crystal structure, the absorption peak around 300 nm was due to the interstitial Zr^3+^ ions^30^. An additional peak around 230 nm in **900A** and **1400A** samples was observed. These shoulder-like absorptions show the existence of additional sub-bands between the primary bands of monoclinic ZrO_2,_ which can be attributed to the band transitions of interstitial O^−^ states to Zr 4d states^31^.

The optical bandgap of the samples was measured by Tauc plot i.e., (αhυ)^n^ versus E from the DRS spectra. The extrapolation of the straight line in the graph to (αhυ)^n^ = 0 gives the value of the energy band gap. The value of **n** is taken as 2 for direct bandgap and ½ for indirect band gap materials respectively. Figure 6(b and c) exemplify the plots of (αhυ)^n^ as a function of E for all the three samples. The **900A** sample exhibited two direct transition regions with band gap values of 4.61 and 2.94 eV. Similarly **1400A** sample also exhibited two direct transition regions with band gap value of 4.85 and 2.78 eV. The **900R** sample has shown direct transition regions with a band gap value of 4.21 and 1.83 eV. However, **900R** sample containing a tetragonal phase has low energy absorption associated with the indirect band transition. Thus, the extrapolation of the linear dependence range between (αhυ)^1/2^ and E (around 4.62–5.21 eV) to (αhυ)^1/2^ = 0, results in an indirect band gap of 3.71 eV.

**Figure 7:**
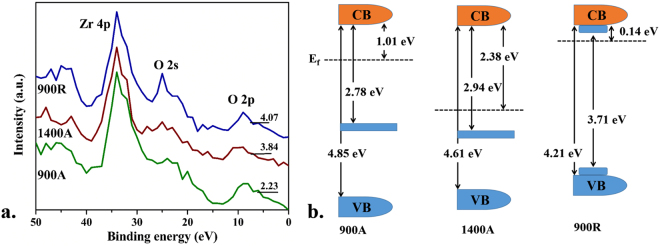
(**a**) Valence band XPS spectra; (**b**) Band structure diagrams of 900A, 1400A and 900R.

Furthermore, the valence band positions (E_v_) were determined from the valence band XPS spectra. Figure 7a represents the valence band XPS spectra of 900 A, 1400A and 900R comprising Zr 4p, O 2 s and O 2p lines. The E_v_ value obtained for **900A**, **1400A** and **900R** were 2.23, 3.84 and 4.07 eV. The conduction band positions (E_c_) were estimated by subtracting the E_v_ values from indirect bandgap value (E_i_) of **900R** and direct band gap value (E_d”_) of **900A** and **1400A**. The E_c_ values calculated for **900A**, **1400A** and **900R** were 2.38, 1.01 and −0.36 eV.

The conduction band edge was very close to the Fermi energy level for **900R** which can be attributed to the significant folding of bands arising from surface defects and oxygen vacancies. The **900R** sample exhibited indirect band transitions due its tetragonal structure which was stabilized under oxygen deficient conditions. Chang *et al*.^32^, also reported that the oxygen vacancies generated by deoxygenation results in the phase stabilization of tetragonal structure. Hence, we attribute the indirect band transition exhibited by **900R** sample to predominantly the interstitial oxygen vacancies. In **900R**, the low valent ions and oxygen vacancies created were sustained due to the absence of oxygen donors and hence results in the stabilization of metastable tetragonal ZrO_2_. Even though the surface hydroxyl groups try to annihilate the oxygen vacancies as the temperature increases, the diffusion rate of hydroxyls will be lower than the rate at which oxygen vacancies are generated. As a result, the metastable tetragonal phase was stabilized under reducing (hydrogen) atmosphere. The presence of defects generated on the surface of **900R** is expected to have played a key role in stabilizing the metastable tetragonal structure. The difference between the direct and indirect band gaps of **900R** was around 0.5 eV which was very high compared to the GW model (0.17 eV) for oxygen free tetragonal ZrO_2_^33^. Thus, this large indirect band gap transition was not from the occupied oxygen vacancy level to the conduction band but from the folded conduction and valence bands in different spatial profiles. In Fig. 7a, the **1400A** sample which was pure monoclinic exhibited an extra small photoelectron peak close to the Fermi energy level which was not observed in other two samples. Morant C *et al*.^34^, also found a similar peak which they attributed to the occupied oxygen vacancy state. These were near to the Fermi level than the O 2p states which could be understood to be a sub-band formation (flat bands) with occupied states that were above valence band. The valence band edges of **900A** and **1400A** samples moved up and also had extended tail formation to decrease the band gap when compared to **900R**. This huge change in band gaps with a difference of 1.67 and 2.07 eV can be due to the Zr^3+^ ions or due to the increase in zirconium content which was confirmed from the EPR and XPS analysis. The formation of sub-bands flat and folded bands in ZrO_2_ can be seen in the band structure diagrams of Fig. 7b.

### Photocatalytic activity measurements

The degradation of methylene blue (MB) dye after exposing it to the visible light in the presence of photocatalyst was analyzed using UV-vis spectroscopy. The photocatalytic mechanism mainly depends on three factors: the ability to absorb photons, efficiency of electrons and holes generated by photo-absorption and their recombination rate. It was believed that the recombination rate of electron-hole pair was reduced due to the presence of Zr^3+^ ions. This fact can be substantiated from the photoluminescence (PL) spectra which clearly depicted the lower recombination rate for **900A** and **1400A** samples (see supplementary information Figure S5). In the case of **900R** sample, it can be understood that oxygen vacancies played a vital role in absorbing visible light which eventually lead to the photocatalytic activity under irradiation of light. The C − S^+^ = C functional group of MB dye (C_16_H_18_ClN_3_S) which has cationic configuration, were attacked by the OH^−^ radicals of ZrO_2_ via coulombic interactions^35^. When the catalyst absorbs photons of energy greater than or equal to its bandgap when exposed to visible/UV radiation, electrons move from valence band to conduction band, creating electron and hole pairs. These electron-hole pairs interact further with surface hydroxyl groups present on ZrO_2_ surface and dissolved oxygen, resulting in superoxide anion radicals (^∙^O_2_^−^) and hydroxyl radicals (^∙^OH). The resultant hydroxyl radicals and superoxide anions act as the oxidizing and reducing agents that help in the complete removal of organic contaminants. Superoxide anions can further react with electrons forming more hydroxyl radicals. The final products formed after degradation of MB dye would be CO_2,_ H_2_O, NH_4_^+^, NO_3_^−^, SO_4_^2−^, Cl^−^^36^.

The reactions taken place in degradation of MB dye are shown below.

$${{\rm{ZrO}}}_{2}+{\rm{h}}\vartheta \to {{\rm{h}}}^{+}+{{\rm{e}}}^{-}$$

$${{\rm{OH}}}^{-}+{{\rm{h}}}^{+}\to {}^{\cdot }{\rm{O}}{\rm{H}}$$

$${{\rm{O}}}_{2}+{{\rm{e}}}^{-}\to {}^{\cdot }{\rm{O}}_{2}^{-}$$

$${\rm{MB}}+{}^{\cdot }{\rm{O}}{\rm{H}}\to {\rm{degradation}}\,{\rm{products}}$$

$${\rm{MB}}+{}^{\cdot }{\rm{O}}_{2}^{-}\to {\rm{degradation}}\,{\rm{products}}$$

**Figure 8:**
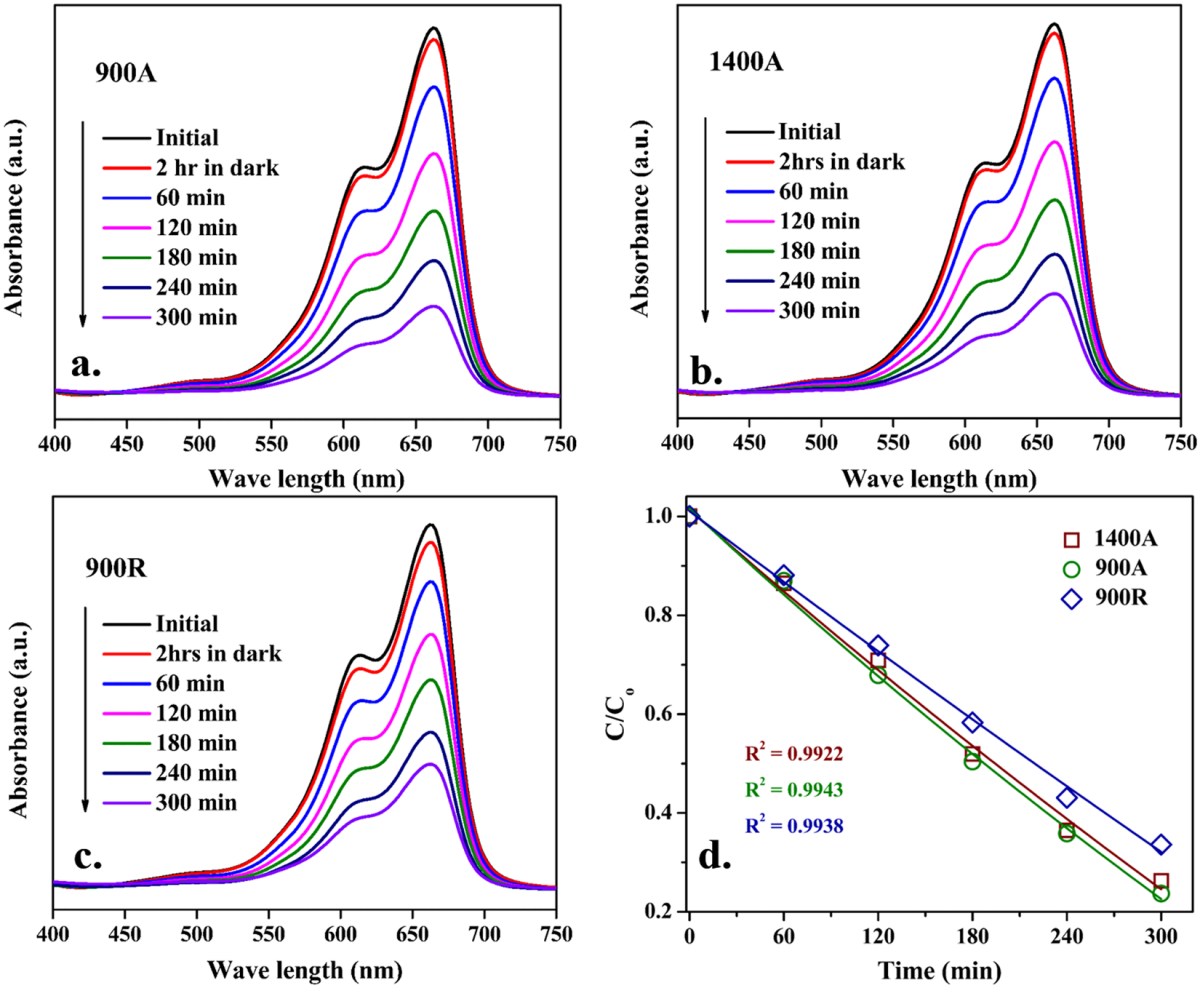
(**a–c**) UV-vis absorption changes of MB dye solution; (**d**) Degradation of MB dye solution over irradiation of time of pyrolyzed ZrO_2_ catalysts.

The absorbance pattern of **900A**, **1400A** and **900R** samples collected from photocatalytic experiments were recorded and shown in Fig. 8(a–c). The absorption curves showed a maxima at a wavelength of 663 nm for all the pyrolyzed ZrO_2_ samples. A continuous decreasing trend with time was observed in the absorption peak value. This leads us to the conclusion that with prolonged exposure to visible light, concentration of MB dye decreases. The percentage degradation reported for **900A**, **1400A** and **900R** were found to be 76, 74 and 66, respectively. The reaction rate constants with their regression values are shown in Table 3 and the decolorization of MB dye from different catalysts can be seen in Figure S6 (see supplementary data). The reaction rate constants were calculated from the slopes of ln(C/C_o_) vs. time plots as shown in Fig. 8d. Interestingly, the reaction rate constant was lower for **900R** black zirconia in contrast to other two white zirconia samples. While the disordered structure is confirmed for **900R** black zirconia samples through Raman spectroscopy along with oxygen vacancies, the abysmal photocatalytic performance is in contrast to what was observed in a recent investigation for black zirconia by Apurba *et al*.^20^. The reason for this will be discussed in the following section. A slight increase in the degradation efficiency associated with the use of **900A** (76%) in contrast to **1400A** (74%) samples possibly could be attributed to availability of more active sites. From the microstructural investigations, it is clear that the crystallite sizes were in the nanometric regime in **900A** samples. The samples exemplified nano-sized pores facilitating reactant access to the surface active sites, thereby creating multiple scattering of light into the pores^37^. Also, the Lewis sites present on the surface of ZrO_2_ possess better adsorption capacity as reported by Wei *et al*.^38^, which is clear from the presence of Zr-OH groups on **900A** in contrast to its limited presence on **1400A** samples.

### Formation of hydroxyl radicals on the surface of catalysts

In order to study the generation of ^∙^OH radicals on the surface of the catalyst, PL spectra were recorded at room temperature. Figures S7(a–d) (see supplementary data) show the variation of intensity in PL spectra (COU solution) under visible light with irradiation time in the presence of a catalyst. It was observed from the spectra that there was a gradual linear increment in PL intensity with increase in irradiation time i.e., from 0 to 60 min at about 490 nm. Hence, it is understood that the generation of ^∙^OH radicals has initiated a reaction between the catalyst and COU solution. It could be inferred that the generation of ^∙^OH radicals on the surface of the catalyst is also proportional to the irradiation time which was also observed by Q. Xiang *et al*. and K. Ishibhasi *et al*.^39–41^. Figure S8 (see supplementary data) indicated that the rate of generation of ^∙^OH radicals, which play a major role in photocatalytic degradation, is high in **900A** compared to other samples.

### Effect of crystal structure on photocatalytic activity

The reduced activity of **900R** black zirconia was primarily attributed to the presence of tetragonal phase around 11%. In order to validate the detrimental effect of the tetragonal phase of zirconia on the photocatalytic behavior, further experiments were performed by stabilizing tetragonal crystal structure in air at lower temperatures. The precursor (zirconium butoxide) was pyrolyzed at 600 and 800 °C in air (referred to as **600A** and **800A**). The **800A** sample showed a mixture of tetragonal and monoclinic phases with 7% tetragonal fraction while **600A** sample predominantly exhibited tetragonal phase of around 87%. X-ray diffractograms of **600A** and **800A** are shown in Figure S9 (see supplementary information). The Raman spectra supported the presence of tetragonal phase in **800A** by exemplifying the presence of B_g_ and A_g_ peaks at 147 and 267 cm^−1^ (see supplementary information Figure S10). The photocatalytic degradation results of methylene blue dye of **600A** and **800A** resulted in 23 and 56% degradation efficiency (see supplementary information Figures S11 and S12) indicating strong deleterious influence of the tetragonal phase. In addition to this, to understand the influence of similar defect chemistry and different crystal structure on catalytic activity, oxygen-deficient black ZrO_2_ was also produced by annealing white ZrO_2_ (**900A**) in reduced atmosphere for 12 hours (referred to as **900R_12**) and was compared with **900R**. The X-ray diffractograms (see supplementary information Figure S13) clearly revealed that the **900R_12** was purely monoclinic in contrast to the monoclinic + tetragonal structure of **900R** sample. The EPR spectra (see supplementary information Figure S14a) showed the presence of oxygen vacancies at g = 2 for both **900R_12** and **900R** samples. The degradation of MB dye solution was carried out in visible light for **900R and 900R_12** (see supplementary information Figure S14b) samples exhibiting a degradation efficiency of 66 and 73%, respectively. This substantiated the detrimental effect of tetragonal crystal structure for the catalytic activity. Furthermore, the exhibition of similar photocatalytic degradation efficiency by **1400A** and **900R_12** proves that the oxygen vacancies (in **900R_12**) played a minimal role in the catalytic activity.

It was also observed from the PL spectra that the intensity of **900R** was higher than that of the **900A** and **1400A** (see supplementary information Figure S6). This indicates that there was a high rate of electron-hole recombination in **900R** samples. Since the X-ray diffractogram of **900R** sample (Fig. 1g) exhibited a mixture of monoclinic and tetragonal phases, it is believed that the monoclinic/tetragonal interface is non-favorable for charge carrier separation. This further validates the reason behind low catalytic activity of **900R** sample. Table 4 (see supplementary information) summarizes the influence of crystal structure and defect chemistry on the photocatalytic degradation of methylene blue dye.

It is clear that the photocatalytic behavior of zirconia depends on various factors such as crystal structure, surface area, surface hydroxyl groups, band-gap, recombination rate and defects^42^. However it is sanguine to assume from the present work that between crystal structure and defect chemistry, the crystal structure has a profound role in the photocatalytic activity of zirconia. Hence, in black zirconia, while the presence of defects assist in increasing the photocatalytic activity, the presence of tetragonal phases on the contrary, even in small amounts can contribute to the opposite effect.

## Conclusions

Zirconia precursor pyrolyzed at various temperatures in air/reduced atmosphere resulted in nanocrystalline white and black zirconia respectively, exhibiting visible light photocatalytic activity towards degradation of methylene blue. Reduction in bandgap resulted in harnessing of light in the visible light regime. White zirconia with pure monoclinic crystal structure exhibited a slight increase in degradation efficiency with the decrease in pyrolysis temperature. This minimal increase in efficiency of white zirconia sample has been attributed to the presence of increased density of nanometric pores (facilitating multiple scattering of light inside the pores) and low recombination rate. Albeit the presence of oxygen vacancies and defect states (from EPR and XPS studies), abysmal performance of black zirconia in contrast to white zirconia towards photocatalytic degradation was ascribed to the presence of tetragonal phases and high recombination rate confirmed by PL measurements. White zirconia containing a mixture of monoclinic and tetragonal crystal structure also provided compelling evidence, further substantiating that crystal structure plays a dominant role in determining the catalytic efficiency in contrast to defect chemistry in the degradation of methylene blue. The black zirconia samples having similar defect chemistry and different crystal structure also authenticated the adverse role of the tetragonal phase in the visible light photocatalytic activity. A suitable photocatalytic mechanism was proposed based on the generation of OH^•^ radicals.

## Experimental Details

### Processing

Zirconium (IV) butoxide (C_16_H_36_O_4_Zr) purchased from Sigma Aldrich, USA was used as precursor to produce zirconia. 8 ml of zirconium butoxide was taken and pyrolyzed in a tubular furnace at a constant heating rate of 5 °C/min in ambient atmosphere (air) at temperatures ranging from 900–1400 °C. The precursor was held at the pyrolysis temperature for four hours and the samples were furnace cooled to room temperature resulting in the transformation of the organic precursor to inorganic zirconia. The ZrO_2_ lumps obtained were crushed using agate mortar and pestle. In addition, ZrO_2_ was also produced by directly pyrolyzing the precursor at 900 °C in reduced atmosphere (i.e., 5% H_2_ + Ar) for comparison. In order to distinctly investigate and discern the influence of crystal size, crystal structure and the role of defects on the photocatalytic behavior, extensive characterization was restricted only to samples processed at 900 and 1400 °C in ambient atmosphere (resulting in white zirconia) and one prototypical sample processed in reduced atmosphere at 900 °C (resulting in black zirconia). The aforementioned samples, hereafter are referred to as **900A**, **1400A** and **900R** in the manuscript indicating the processing temperature and the atmosphere (**A** refers to ambient and **R** refers to reduced atmosphere).

### Structural characterization and spectroscopic measurements

The thermogravimetry (TG-DTG) measurements were carried for zirconium butoxide in an alumina crucible from 25–1300 °C at a heating rate of 20 °C/min in air by NETZSCH STA _449_ F_3_ Jupiter. The functional groups of the samples were determined in the Perkin Elmer Spectrum_1_ FTIR instrument. The powder samples were mixed with KBr and pelletized subsequently. The pellets were analyzed in the transmission mode at a scan range of 4500–400 cm^−1^. X-ray diffractograms were obtained from the PANalytical diffractometer performed in the 2θ range of 10–90° with a step size of 0.15 using Cu K_α_ radiation. Raman spectroscopy was done using LabRAM HR 800 instrument at a wavelength of 632 nm at room temperature in 600 grating.

Scanning electron microscopy (SEM) images were taken in Hitachi S4800 FE SEM with tungsten thermionic gun in secondary electron (SE) mode. To minimize the charging effects the samples were sputter-coated with gold for 120 seconds prior to imaging. Transmission electron microscopy (TEM) was done using Tecnai microscope at an accelerating voltage of 200 kV. The powder samples were ultrasonicated in ethanol solution for 30 min and then a few drops were placed on the carbon-coated copper grids for TEM studies. The nitrogen adsorption-desorption isotherms of the samples were measured at −196 °C using Micromeritics TriStar II after degassing the samples for six hours at 150 °C. The specific surface area, *S*_BET_ was calculated using Brunauer-Emmett Teller (BET) equation.

The diffuse reflectance spectra (DRS) in absorbance mode were recorded on an Evolution 220 UV-Visible Spectrophotometer from Thermo Fisher Scientific. The optical bandgap of the samples was measured by plotting **(αhυ)**^**n**^ versus **hυ**. The X-ray photoelectron spectroscopy (XPS) was performed for samples using Omicron NanoTechnology Inc,. Electron Paramagnetic Resonance Spectrometry (EPR) was done on JEOL model JES FA_200_ instrument at room temperature using X band frequency of 9.65 GHz with a sensitivity of 7*10^9^ spins/0.1 mT. The recorded intensity values were plotted against magnetic field (G) and the corresponding **g** values were calculated. Photoluminescence studies were performed on the Horiba Scientific FluoroMax-4 Spectrofluorometer at room temperature using emission spectra excited at a wavelength of 235 nm with a slit 1 nm. The visible light photocatalytic activity of catalysts was evaluated by detecting the hydroxyl radical formation using 0.1 mM of coumarin (COU) solution. 40 ml of this solution was taken with 100 mg of the catalyst and was kept in dark to reach an adsorption-desorption equilibrium prior to visible light irradiation. A 500 W Xenon lamp was used as visible light source and a 2.5 ml of sample was taken for every 15 minutes, centrifuged to measure the PL intensity excited at 340 nm.

### Photocatalytic degradation of methylene blue

The photocatalytic activity of samples was investigated in visible wavelength using Heber immersion type photoreactor using 500 W Xenon lamps as the light source. 50 mg of ZrO_2_ powder sample was dispersed in 100 ml of methylene blue (MB) solution of 0.03 mM concentration. The solution was continuous stirred and a highly polished aluminum reflector was used to focus the light on the dye solution containing ZrO_2_. The light source was continuously cooled by water circulation through an outer jacket. A small cooling fan was also provided at the bottom of the reactor. The solution was kept in a dark atmosphere for two hours to ensure adsorption-desorption equilibrium between the sample and solution. The solution was then irradiated with visible light for five hours. During the exposure, 1.5 ml of MB solution was regularly taken out at time intervals of 30 minutes. The solution was centrifuged to remove any remnant solid powder particles. The collected samples were further analyzed with UV-vis spectrophotometer to study their adsorption characteristics and degradation behavior. The percentage decolorization was calculated using following equation:

1 $$ \% \,{\rm{decolorization}}=({{\rm{C}}}_{{\rm{o}}}-{\rm{C}})/{{\rm{C}}}_{{\rm{o}}}\ast 100$$

where, C_o_ and C were the initial (before illumination of visible light) and final concentration of the solution in mg/L. The photocatalytic reaction rate after adsorption-desorption equilibrium was given by the Langmuir-Hinshelwood model:

2 $$\mathrm{ln}({\rm{C}}/{{\rm{C}}}_{{\rm{o}}})=-{\rm{Kt}}$$

where, K is the reaction rate constant and C_o_ and C were the concentration at time t = 0 and t = t respectively. The reaction rate constant was calculated from the slope.

## Electronic supplementary material

Supplementary Information
