# Supplementary material for: Dominating role of crystal structure over defect chemistry in black and white zirconia on visible light photocatalytic activity
Source: Sci Rep. 2018 Apr 3;8:5541. doi: 10.1038/s41598-018-23648-0 (PMC5882956; doi:10.1038/s41598-018-23648-0)
Supplement: Supplementary file 1 — Supplementary Information [file 41598_2018_23648_MOESM1_ESM.docx]

**Supplementary Information**

**Dominating role of crystal structure over defect chemistry in black and white zirconia on visible light photocatalytic activity**

Sri Ramya Teeparthi, Eranezhuth Wasan Awin, Ravi Kumar^*^

*Laboratory for High Performance Ceramics, Department of Metallurgical and Materials Engineering, Indian Institute of Technology Madras*

*(IIT Madras), Chennai-600036, India.*

*Corresponding author:

Email: [nvrk@iitm.ac.in](mailto:nvrk@iitm.ac.in)


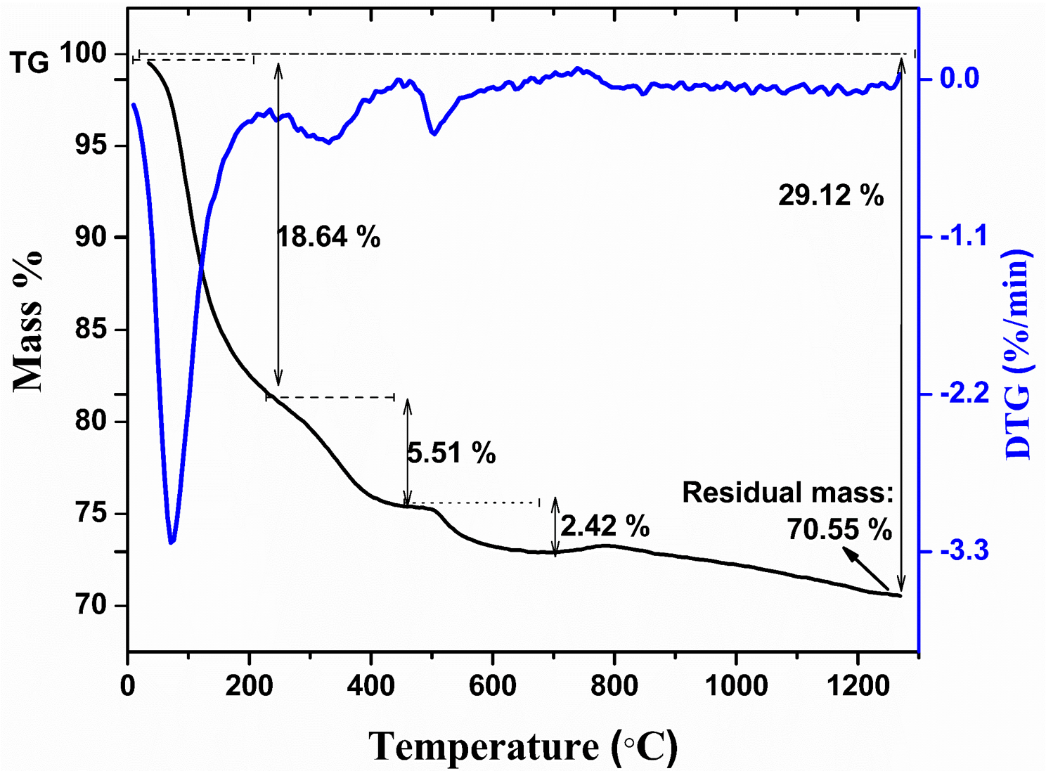


Figure S1. TGA of zirconium butoxide performed from 25 to 1300 ^°^C at a heating rate of 20 ^°^C/min in ambient atmosphere.


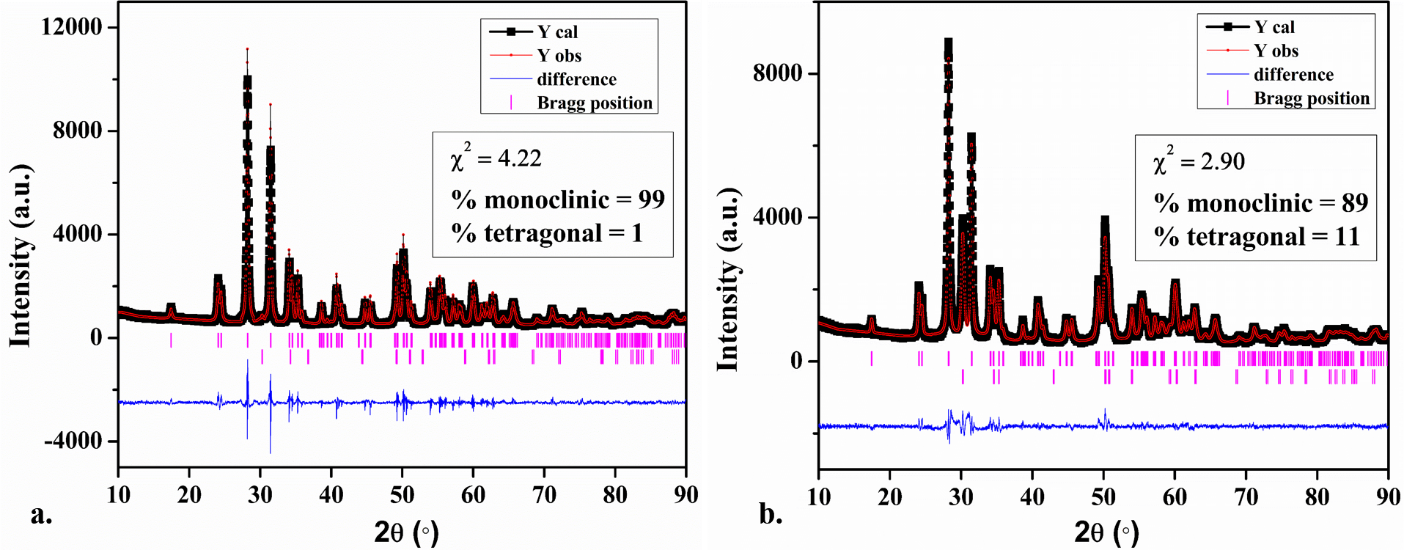


Figure S2. Rietveld refinement of the XRD data of **a**) 900A (White) and **b**) 900R (Black) zirconia samples





Figure S3. Nitrogen adsorption-desorption isotherms of pyrolyzed samples.

Table 1. Textural properties of catalysts from N­­_2_ isotherm analysis.

| **Parameter** | **900A** | **1400A** | **900R** |
| --- | --- | --- | --- |
| **BET Surface area (m^2^/g)** | 1.1095 | 0.951 | 0.639 |
| **BJH adsorption avg pore size (nm)** | 55 | 20 | 10 |


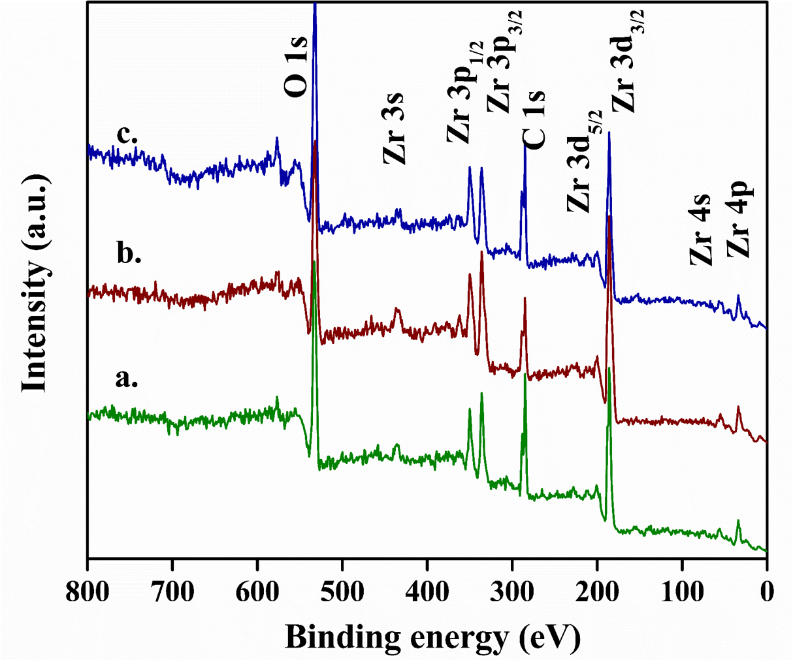


Figure S4. XPS wide scan spectra of **a**) 900A (White), **b**) 1400A (White) and **c**) 900R (Black) showing different regions of zirconia.

Table 2. Values of percentage relative areas of Zr-OH, Zr-O-C, Zr^4+^ and Zr^2+^

| **Sample** | **Zr - OH** | **Zr – O - C** | **Zr^4+^** | **Zr^2+^** |
| --- | --- | --- | --- | --- |
| **900A** | 53.1 | 16.3 | 8.4 | - |
| **1400A** | 39.3 | 11.6 | 13.4 | - |
| **900R** | 53.6 | 14.9 | - | 10.0 |

Table 3. Values of percentage degradation, reaction rate constants and R^2^.

| **Sample** | **% deg** | **K , (/ min) *10^-4^** | **R^2^** |
| --- | --- | --- | --- |
| **900A** | 76 | 7.96 | 0.9943 |
| **1400A** | 74 | 6.15 | 0.9922 |
| **900R** | 66 | 3.15 | 0.9938 |

**
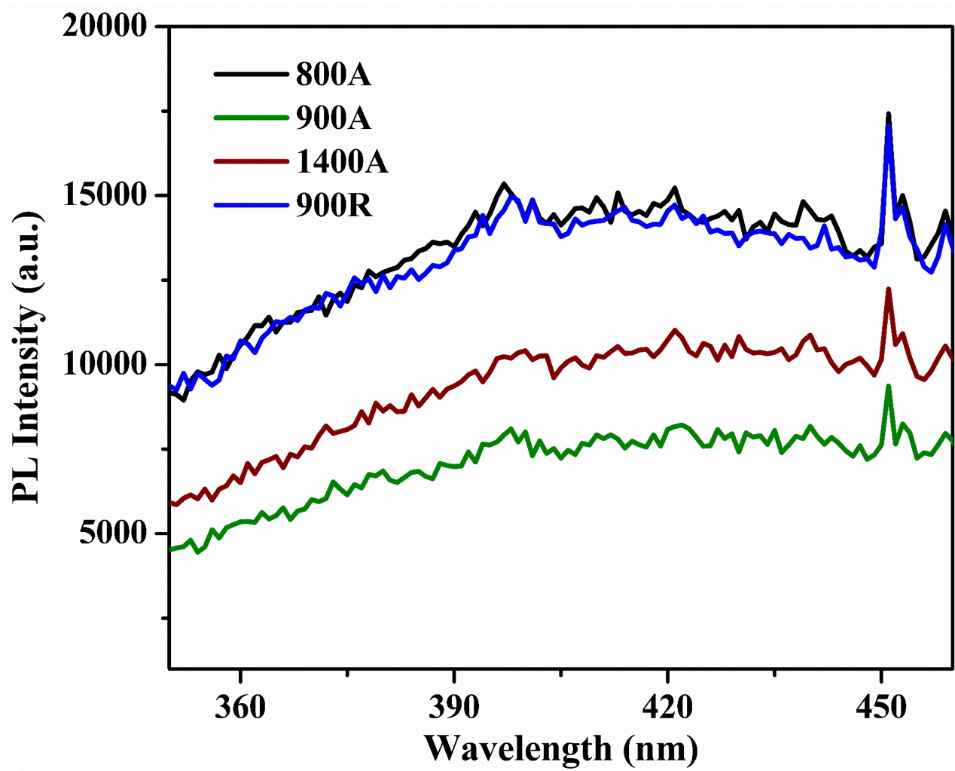
**

Figure S5. PL spectra taken from the solid film of pyrolyzed catalysts using excitation wavelength of 235 nm with slit 1 nm.


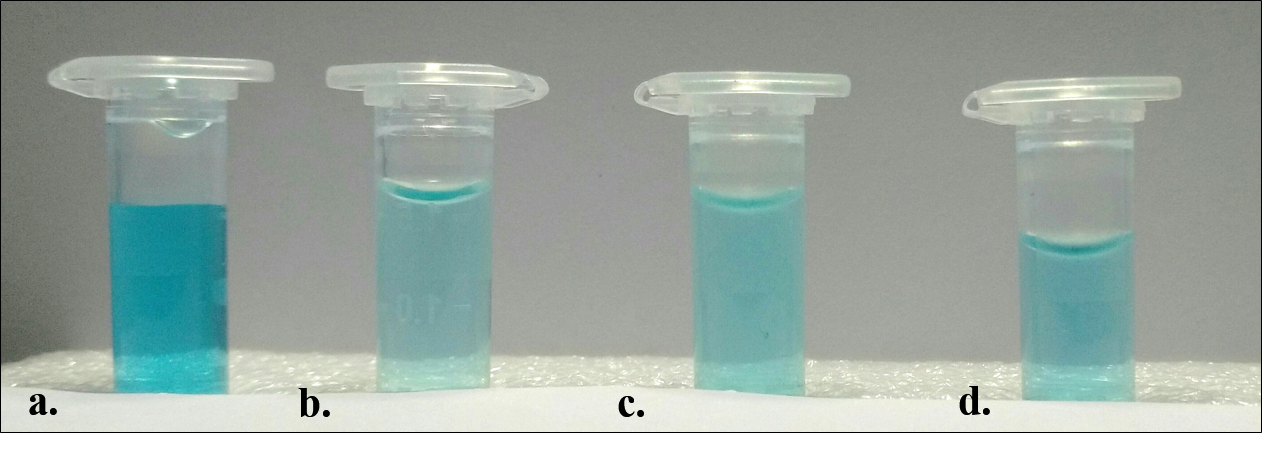


Figure S6. Decolorization of MB dye solution **a**) Initial, **b**-**d**) irradiation of light after 300 min for 900A (White), 1400A (White) and 900R (Black) zirconia samples.


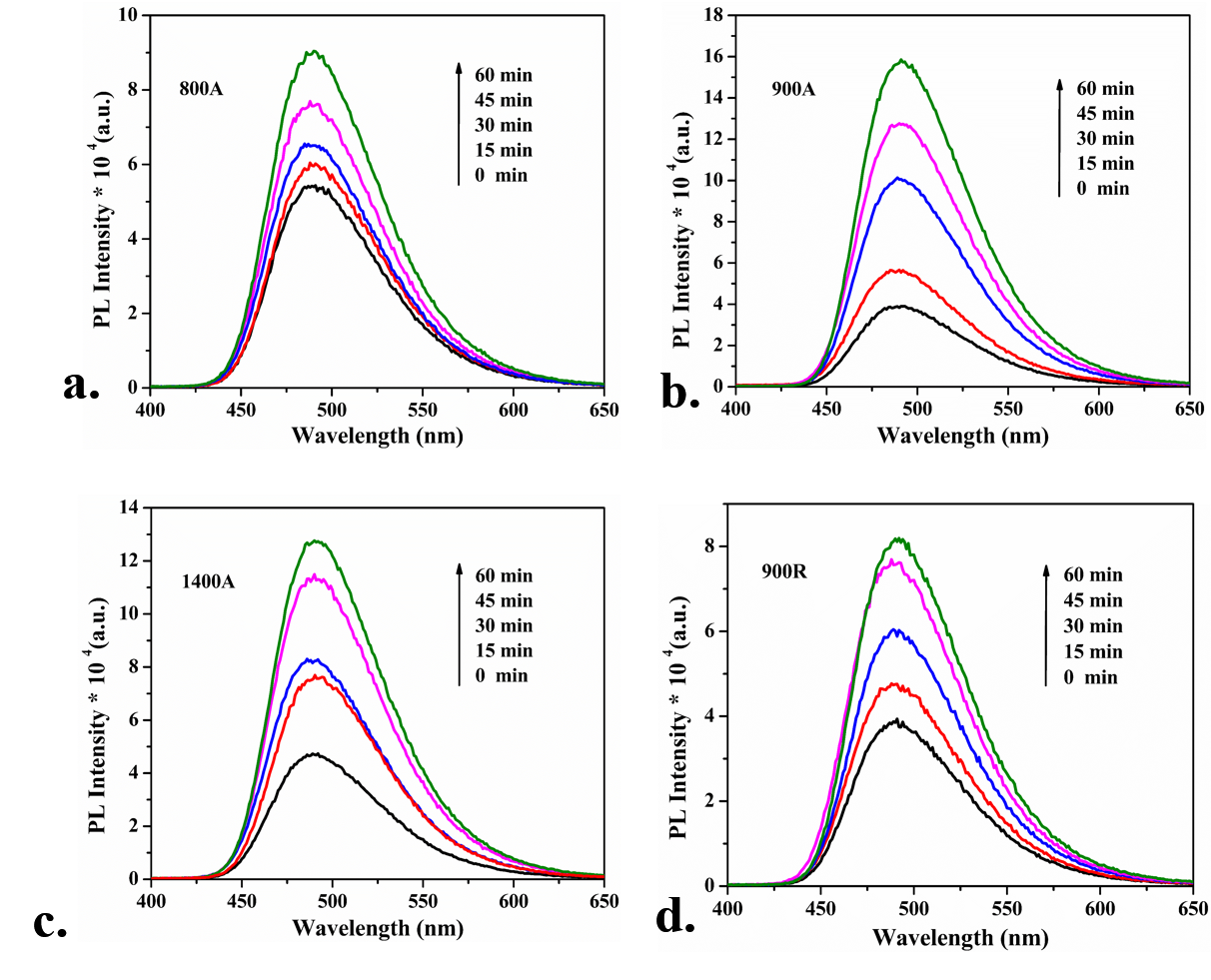


Figure S7. (**a-d**) PL spectral changes observed for the catalysts prepared during illumination over irradiation time with excitation wavelength of 340 nm with slit 1 nm in COU aqueous solution


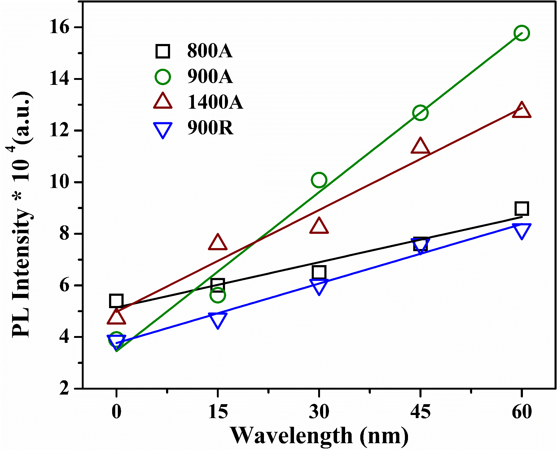


Figure S8. Linear plot of PL intensity versus wavelength to calculate the rate of hyroxyl radicals fromation.





Figure S9. X-ray diffractograms of pyrolyzed zirconium butoxide at 800 and 600 °C in ambient atmosphere (where, M refers to monoclinic and T refers to tetragonal).

**
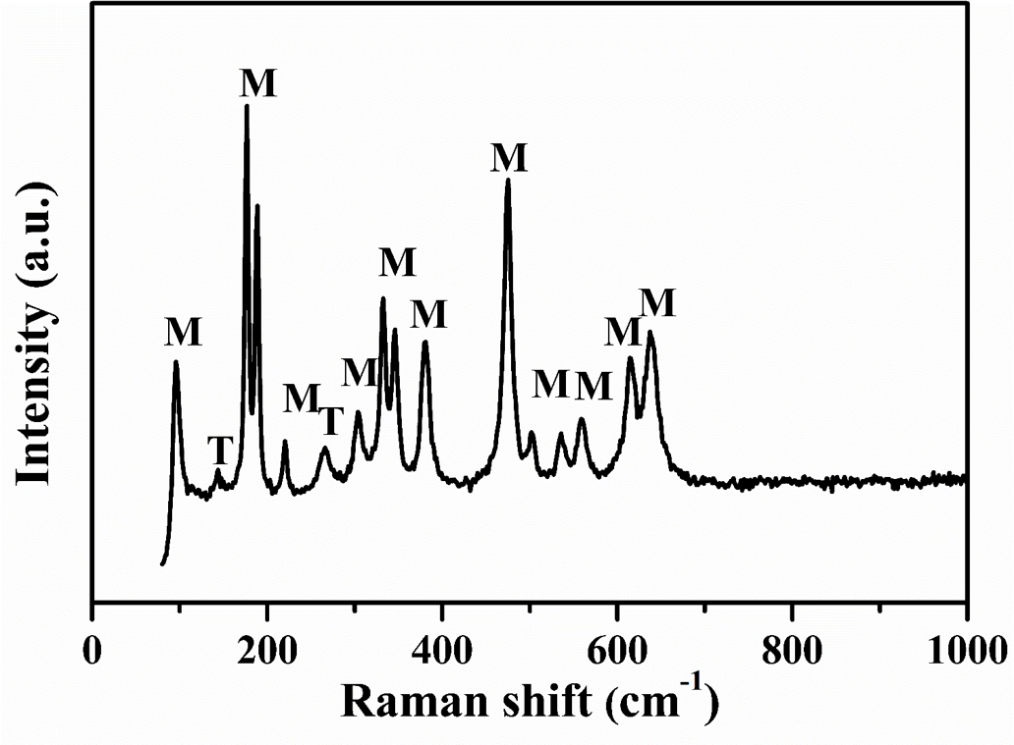
**

Figure S10. Raman spectra of 800A sample showing the presence of both tetragonal and monoclinic bands.

**
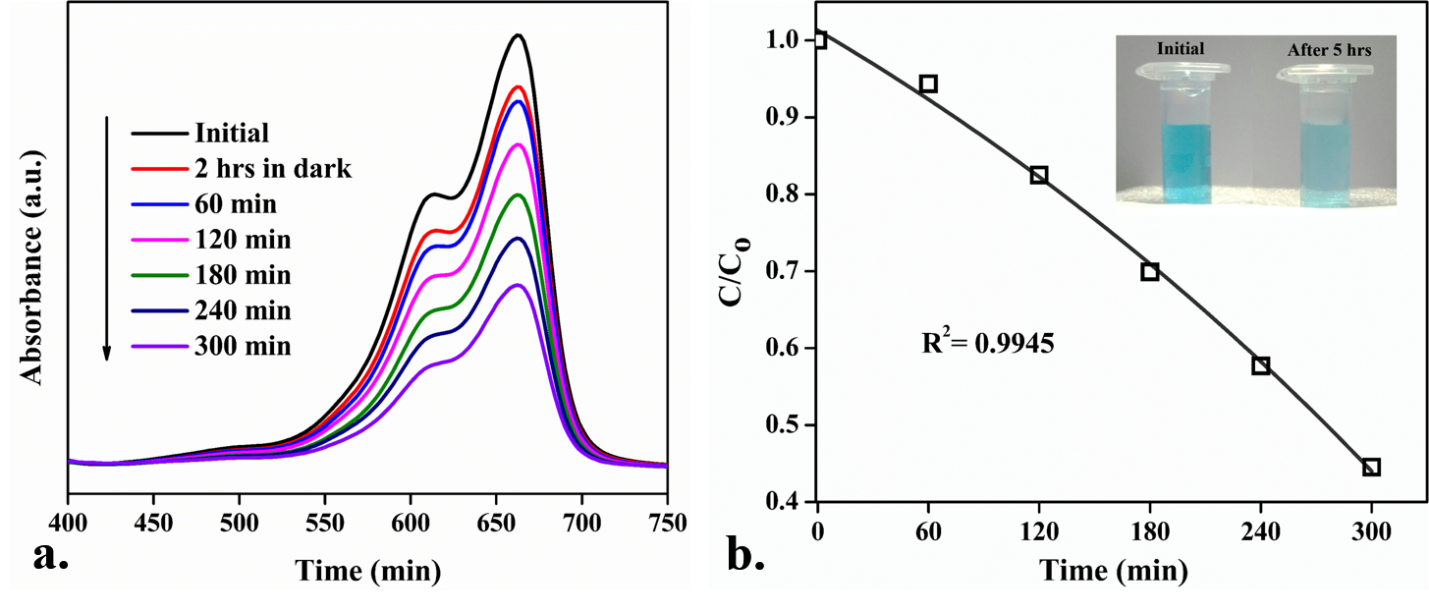
**

Figure S11. **a**) UV-vis absorption changes of MB dye solution of 800A sample; **b**) Degradation of MB dye solution over irradiation of time with 800A sample with percentage efficiency of 56 %. The inset shown in figure is the decolorization behavior before illumination of light and after five hours.


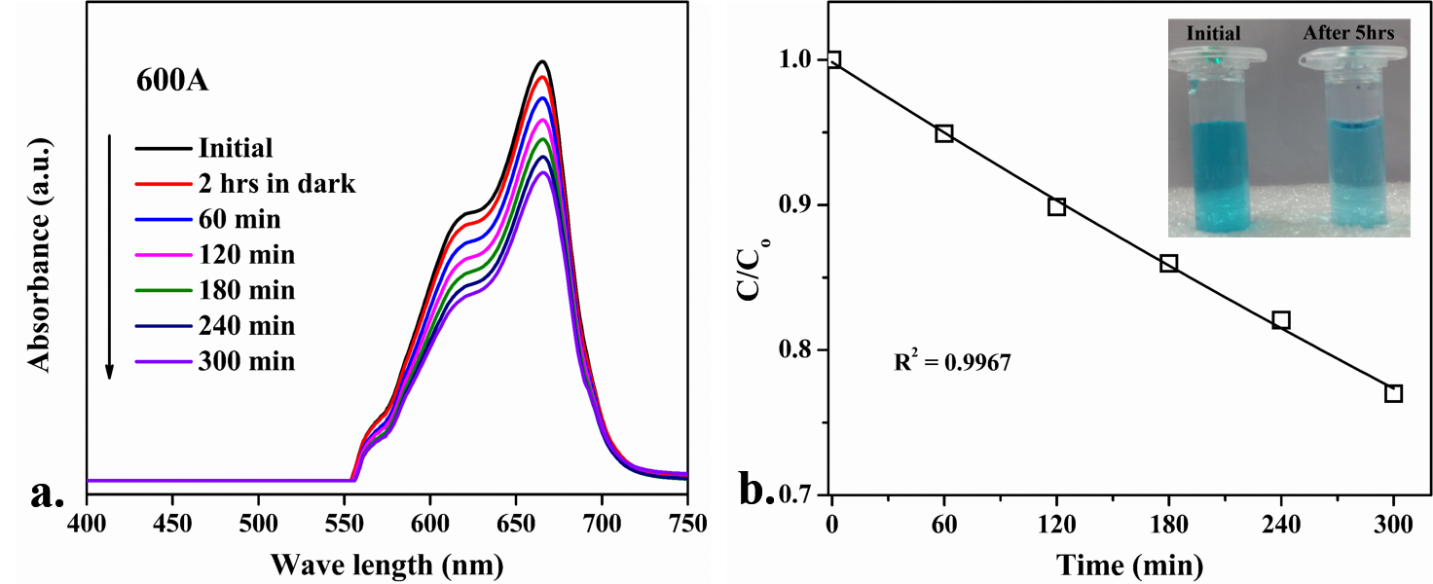


Figure S12. **a**) UV-vis absorption changes of MB dye solution of 600A sample; **b**) Degradation of MB dye solution over irradiation of time with 600A sample with percentage efficiency of 23 %. The inset shown in figure is the decolorization behavior before illumination of light and after five hours.

*

*

Figure S13. X-ray diffractograms of 900R and 900R­_12 samples illustrating the differences in crystal structure. The 900R sample shows a mixture of monoclinic and tetragonal phase whereas 900R_12 shows pure monoclinic crystal structure (where, M refers to monoclinic and T refers to tetragonal).

*
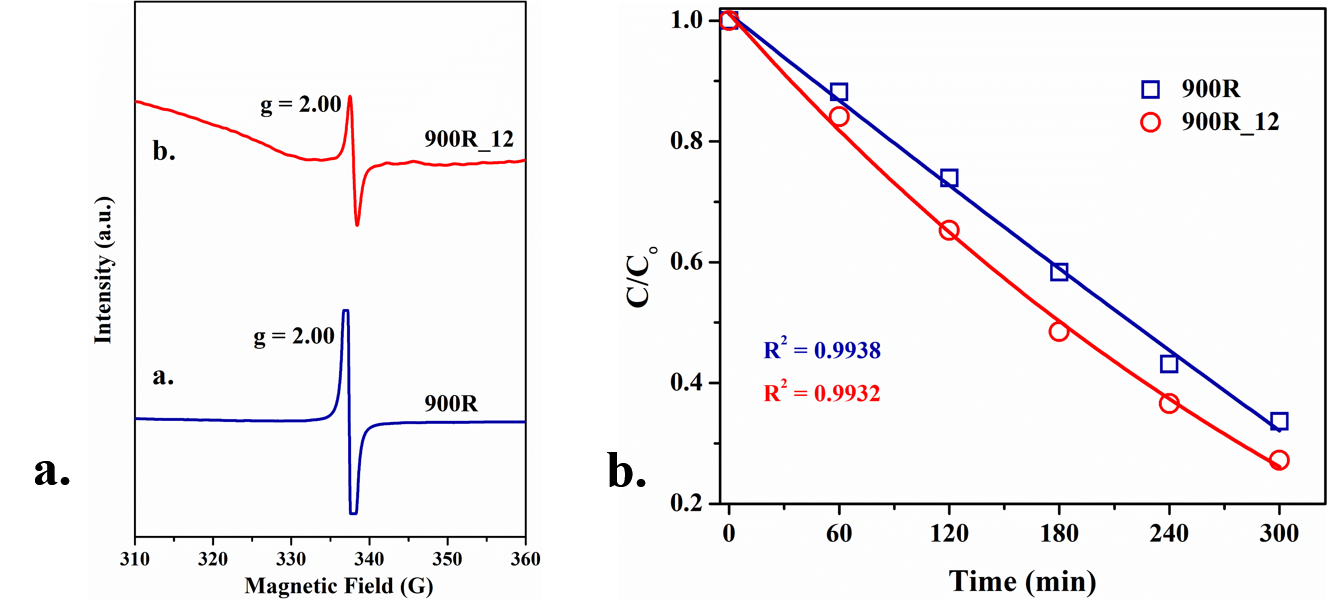
*

Figure S14 **a**) EPR spectra of 900R and 900R_12 samples showing presence of oxygen vacancies at g = 2 **b**) Degradation of MB dye solution over irradiation of time of 900R and 900R_12 samples with degradation efficiency of 66 and 73 %.

Table 4. Experimental work done and results obtained.

| **S.No.** | **Sample** | **Ambience;**  **Temperature** | **Crystal structure** | **Defect chemistry** | **% degradation efficiency** |
| --- | --- | --- | --- | --- | --- |
| 1 | **900A** | Air; 900 °C | Monoclinic | - | 76 |
| 2 | **1400A** | Air; 1400 °C | Monoclinic | - | 74 |
| 3 | **900R**  (black) | Reduced; 900 °C | Monoclinic +  Tetragonal (11%) | Oxygen deficient | 66 |
| 4 | **800A** | Air; 800 °C | Monoclinic + Tetragonal (7%) | - | 56 |
| 5 | **600A** | Air; 600 °C | Monoclinic + (87%) Tetragonal | - | 23 |
| 6 | **900R_12**  (black) | Reduced; 900 °C | Monoclinic | Oxygen deficient | 73 |
